# Users' Feedback on COVID-19 Lockdown Documentary: An Emotion Analysis and Topic Modeling Analysis

**DOI:** 10.3389/fpsyg.2022.944049

**Published:** 2022-06-28

**Authors:** Xiaochuan Shi, Miaoyutian Jia, Jia Li, Quiyi Chen, Guan Liu, Qian Liu

**Affiliations:** ^1^School of Cyber Science and Engineering, Wuhan University, Wuhan, China; ^2^School of Journalism and Communication, Jinan University, Guangzhou, China; ^3^International School, Jinan University, Guangzhou, China; ^4^Research Center of Computational Journalism and Communication, Jinan University, Guangzhou, China

**Keywords:** COVID-19, health measures, LDA topic modeling, lockdown, emotion analysis

## Abstract

Conducting emotion analysis and generating users' feedback from social media platforms may help understand their emotional responses to video products, such as a documentary on the lockdown of Wuhan during COVID-19. The results of emotion analysis could be used to make further user recommendations for marketing purposes. In our study, we try to understand how users respond to a documentary through YouTube comments. We chose “The lockdown: One month in Wuhan” YouTube documentary, and applied emotion analysis as well as a machine learning approach to the comments. We first cleaned the data and then introduced an emotion analysis based on the statistical characteristics and lexicon combination. After that, we applied the Latent Dirichlet Allocation (LDA) topic modeling approach to further generate main topics with keywords from the comments and visualized the distribution by visualizing the topics. The result shows trust (22.8%), joy (15.4%), and anticipation (17.6%) are the most prominent emotions dominating the comments. The major three themes, which account for 70% of all comments, are discussing stories about fighting against the virus, medical workers being heroes, and medical workers being respected. Further discussion has been conducted on the changing of different sentiments over time for the ongoing health crisis. This study proves that emotion analysis and LDA topic modeling could be used to generate explanations of users' opinions and feelings about video products, which could support user recommendations in marketing.

## Introduction

Conducting emotion analysis and generating users' feedback from social media platforms could significantly help to understand users' emotional responses to video products, and referring to the results of emotion analysis is very important to optimize the recommendation system. A further customized strategy could be suggested to improve the marketing. This study chose a documentary on the lockdown of Wuhan during COVID-19 as an example, to explore the application of users' emotion analysis in marketing.

In December 2019, early COVID-19 cases were reported from Wuhan city, in Hubei, China. More diagnosed patients were soon detected in other cities in China and around the globe. Physicians and pathologists initially reported it as “atypical pneumonia”, which means another type of pneumonia that is not caused by the three most common pathogens. Wuhan, as first city to report COVID-19 cases, went through a lockdown as a health measure on 23 January 2020. After several samplings and laboratory analyses, the pathogen was identified and named COVID-19 by the WHO on February 11, 2020 (Organization WH, 2020a). The virus was named “SARS-CoV-2” by the International Committee on Taxonomy of Viruses (Gorbalenya et al., 2020). As one of the most stringent health and medical health measures in human history, Wuhan's lockdown has triggered a lot of discussion around the world. Users' feedback, especially emotional responses to this health measure, has drawn attention as well.

In order to better communicate with the users, city promotional films or city documentaries are often used to showcase regions' features or important events. The documentary we chose is about lockdown in Wuhan; it showcases the special lockdown period of Wuhan in detail. The Chinese National Health Commission (NHC) announced over 15,000 death and around 860,000 confirmed COVID-19 cases in China at the end of April 2022. Wuhan's lockdown in response to the COVID-19 pandemic showed significant results as a public policy at the city level. Shanghai, Xi'an, and other cities in or outside China have adopted this method as well at different levels to prevent the spread of the virus. Besides lockdown of an area, masking, and social distance, vaccination has proven to be effective and is recommended by scientists, such as Harry Stevens who simulated the COVID-19 infection, pointing out the importance of social distancing as an effective method of prevention (14 March 2020) (China NHCotPsRo, 2020).

Understanding the users' emotional responses to video products and getting to know their feedback on the lockdown of a city could be done by analyzing comments on the documentary “Wuhan War Epidemic”, a 33-min documentary published by CGTN (China Global Television Network) about the whole process of Wuhan's lockdown as a health measure. This documentary records in detail how the epidemic went from a rapid outbreak to a gradual containment. It also records the great sacrifice and dedication of front-line citizens, such as medical staff, community workers, volunteers, couriers, and other ordinary Wuhan citizens who keep working in the closed city and fight the epidemic together, which has triggered emotional feedback from users.

Referring to the results of sentiment analysis is very important to optimize the recommendation system. Recommendation systems are often used in marketing to recommend which products are of interest to users. However, the results of general search-based recommendation feedback are difficult to match to the specific needs of users, because the general system is not tailored for each different search user. For example, a 10-year-old and an adult wanting to query “best movies of 2021” will get the same set of movies, but obviously, they need different movies. Therefore, sentiment analysis is of vital importance to optimize the recommendation system.

Given the desire to know what the audience thinks about the video, especially their emotional responses, this article focuses on the comments on this documentary on the YouTube website, conducts an emotion analysis, and extracts the main topics with the LDA topic modeling approach. Analyzing and extracting knowledge from these responses from citizens could improve the city's video products, and provide suggestions for future user recommendations in marketing.

## Literature Review

Numerous researchers used emotion analysis as a popular method in text analysis (Settanni and Marengo, 2015). It is applied in various fields including the COVID-19 pandemic and users' preferences. For example, to understand what people think of food, researchers such as M. J. Widener focused their research on American people's attitudes toward food, including nutritious food and harmful food (Widener and Li, 2014). These users' text data were collected from different areas in America. Text data could be collected from the news or social media, such as tweets, Facebook, YouTube, etc. For example, L. Pépin, and P. Kuntz collected over 70,000 pieces of data from tweets to investigate the information from clients who had brought products from a French Company (Pepin et al., 2017).

Emotion research and analysis could be used to improve recommended system performance. Some scholars studied how to provide better recommendations by incorporating users' interests as well as emotional analysis results (Jaiswal et al., 2019). Some studies focused on emotion and social norms that influence the users' decisions and used socio-cognitive agent-based simulations for recommending marketing strategies (Schaat et al., 2015). The collation of data is not only limited to text input; some researchers used wearable physiological sensors to collect data and built an emotion-based music recommendation system (Ayata et al., 2018). Getting the emotional responses and generate users' opinions from text-based data in user negotiation or communication could improve user recommendations for marketing purposes, so scholars strived in many approaches, including emotion analysis and topic modeling on many platforms, with some even using a hybrid AI(artificial intelligence) model, to predict new COVID-19 cases (Zheng et al., 2020).

In addition to understanding people's feelings when responding to video products, another approach to generate users' feedback as readable explanation is Latent Dirichlet Allocation (LDA) topic modeling, where the machine learning approach could help process data to get people's major discussion topics on the public health measure. Scholars applied LDA topic modeling to collect Twitter data sets about COVID-19-related data (Chen et al., 2020).

## Methods

As COVID-19 spreads rapidly, the YouTube platform, a new media video service provider, has been an active arena for health communication on COVID-19, such as its new measures, policies, symptoms, treatment, pathogenesis, spread, patient story, and prevention. Documentaries have played an important role in health communication during the epidemic, but there are few relevant studies on this issue. Many researchers focus on the content and shooting technique but there is a lack of analysis on its users' emotional feedback. “The lockdown: One month in Wuhan” documentary showing Wuhan under the epidemic situation has had a profound influence. Therefore, we applied emotion analysis as well as topic modeling to the comments of this documentary in order to understand the users” responses to this video product.

In this study, we collected user comments, cleaned the data and analyzed the comments with Python software 3.6, and conducted emotion analysis as well as an LDA topic modeling approach to generate main topics with keywords from the comments. After that, we visualized the distribution by plotting the topics into two-dimensional planes to further analyze the relationships between topics. We tried to understand what people think of the video products through an analysis of health communication data collected from the YouTube platform. The recommendation system could be improved by incorporating the emotion analysis result in marketing.

### Choosing the Documentary

The reason why “The lockdown: One month in Wuhan” documentary was chosen is because of its influence and relevance. The documentary was broadcasted on the CGTN English Channel on February 28, and it was also posted on CGTN's official website, app, and CGTN's overseas social platform accounts (YouTube, Twitter, and Facebook). Once the film was broadcasted, it attracted a great deal of attention from overseas media. It was successively received by 165 companies in 21 countries and regions, including the American Broadcasting Corporation (ABC), British Channel 4 TV, French TV5 MONDE, Italian TGCOM24, Canadian Broadcasting Corporation, and Japan's Asahi TV. The adoption of overseas TV channels and new media platforms has received an enthusiastic response. As of October 4, the documentary has more than 16,000 video interactions on Facebook accounts and more than 1,000 comments. It has been reposted by the official accounts of the Permanent Mission of the People's Republic of China to the United Nations, the Chinese Embassy in the UK, and the Chinese Embassy in Germany on Twitter.

The reason for choosing the YouTube platform is that it is one of the most popular free video-sharing sites for internet users worldwide. Video views show how many times this video has been watched, while videos rated with likes or with comments by users would demonstrate what users think of the corresponding video or comments. On the YouTube platform, this documentary has the highest view in videos with “Wuhan” as a keyword that appears in the video title. Until 3 October 2020, “The lockdown: One month in Wuhan” received over 180,000 likes, 28,630 comments, and had been watched 17,745,428 times on YouTube since its first launch on 28 February 2020.

### Data Collection and Processing

We looked into the audiences' comments under the “The lockdown: One month in Wuhan” documentary video on the YouTube platform and collected the 1,560 most popular comments ranked by YouTube. Then we removed the non-English comments and sorted out 1,274 English comments only.

Details of the processing of the data were listed in Supplementary Figure 1, as shown; we first cleaned comments' text data with Python 3.6 by removing English common stop words, such as “the”, “this”, “them”, and “they”.

Then this study conducted an emotion analysis on YouTube comments, to get the users' emotional responses to the lockdown of a city. We used the NRC Emotion Lexicon, created by the National Research Council Canada for emotion analysis. It was the authoritative Emotion Lexicon commonly used by the international academic community, which showed eight basic emotions, namely anger, fear, anticipation, trust, surprise, sadness, joy, and disgust, as well as negative and positive emotions (Inkpen and Strapparava, 2010; Mohammad, 2013). We visualized the result in Supplementary Figures 2, 3.

To help generate topics from texts, there are two main topic model methods: Probabilistic Latent Semantic Analysis (PLSA) and LDA, wherein LDA is commonly used in the fields of text mining (HEROPHEC, 2020), health studies (Organization WH, 2020b), health education (Ming et al., 2020), and detecting patients (WiseNews, 2020) studies. LDA is a generative statistical model that assumed each word in a document is representing a topic with a certain probability, and with a three-level hierarchical Bayesian model, we analyzed this combination of words belonging to different topics (Hassanpour and Langlotz, 2016). Then we sought different topics through the use of Gibbs Sampling techniques (Goyal and Gomeni, 2013). LDA was applied to process the data in our study.

We first prepared a document-term matrix (DTM) and used TF-IDF for further LDA modeling analysis to derive different topics. To conduct the LDA topic modeling, we then needed to find a topic number that contributed to the improvement of the topic's semantic understanding. A large topic number might result in uninterpretable results (Kandula et al., 2011), whereas a small number could result in not sufficiently separating the topics. As a result, we chose 10 topics to analyze with the help of Python version 3.6 and the LDAvis tool (Hassanpour and Langlotz, 2016). We set λ = 1 and topic number = 10 to conduct LDA analysis (Li et al., 2015) and got 10 groups of topics' keywords as an outcome; then we generated the topic names according to the corresponding keywords as shown in Supplementary Table 1.

Utilizing the LDAvis tool, we also conducted a visualization, where each opinion topic was represented as a cycle in Supplementary Figure 4. The length of the cycle centers' distance represents the topic distance and the size of the cycle was determined by the overall prevalence of the topic (Li et al., 2015; Hassanpour and Langlotz, 2016). To further analyze the topics, they were divided into six different main themes, shown in Supplementary Table 1.

## Results

For emotion analysis, Supplementary Figure 2 shows the proportion of all kinds of emotional word frequency. It suggests different emotions evolve globally toward the lockdown in Wuhan, among which trust (22.8%), joy (15.4%), and anticipation (17.6%) are the most prominent ones dominating in the comments. Supplementary Figure 3 shows the distribution of emotional word frequency over time. The results show that the time distribution peaks of all kinds of word frequencies are concentrated in April and September.

For topic modeling analysis, we have set 10 as the topic number and generated corresponding keywords. Supplementary Figure 4 illustrates the topic allocation results, in which 10 different topics for users' feedback are distributed as separated or overlapped circles. As shown in Supplementary Figure 4, PC1 and PC2 represents the transverse and longitudinal axis respectively, while the length of the cycle centers to represent the topic distance were demonstrated through multidimensional scaling in the two-dimensional plane (McLaurin et al., 2014), and the size of the cycle is determined by the overall prevalence of the topic (Li et al., 2015; Hassanpour and Langlotz, 2016). Supplementary Figure 5 presents the top 30 relevant terms for topic 6. Supplementary Table 1 specifically shows six themes and ten corresponding topics with the data analysis, and the percentage represents the proportion of this topic overall. These topics were categorized into themes according to the analysis of the keywords. The top two themes were about Chinese fighting COVID-19 (accounting for 30.1%) and medical workers (accounting for 22.8%).

## Discussion

During the spread of COVID-19, “The lockdown: One month in Wuhan”, as one of the first YouTube documentaries about Wuhan's lockdown, offered us a window to discover what had happened, and as a social media platform, it also served as an arena for emotion and opinion expression.

To get the emotional responses and to generate users' feedback, we combined the results of emotion analysis and topic modeling. We observed a positive attitude and anticipation for the future in comments toward this health measure. When emotion analysis helped to understand users' responses emotionally, the topic modeling approach confirms some of the emotions with further details. As can be seen from Supplementary Table 1, the topics that netizens were most concerned about were “the Chinese fight against COVID-19” (30.1%), “medical workers” (22.8%), “Respecting lockdown (19.8%), and “Praying for safety” (14.2%).

Health measures on this global epidemic have aroused great social attention. The first emotional peak for public opinion was reached in April and Wuhan, as the epicenter in China, began to spread the virus wildly, the local story of fighting the epidemic attracted a large audience. Emotion analysis showed trust (22.8%), joy (15.4%), and anticipation (17.6%) are the most prominent with the first peak in April. Supplementary Figure 3 described the estimated term frequency within topic 6 “Wuhan story”. As shown in Supplementary Figure 4, the term “Wuhan” ranks first, followed by “great”, “thanks”, and “city”, which meant netizens focused more on expressing gratitude after watching the Wuhan story during the early period.

For Supplementary Figure 6 (left), we were informed that topic two “Pray for safety” was the most popular topic, taking up to 14.2% of discussion, with the top-30 relevant term for Topic 2 listed on the right side. It showed “pray”, “strong” and “together” as having the highest proportion respectively. We inferred that the netizens showed strong support for all the people in Wuhan.

Users' feedback, especially emotional responses on lockdown, a health measure that violates personal freedom, is controversial in many countries. A growing trend of all emotions was observed since July 2020, where “anger” and “disgust” grew faster, while “joy” and “surprise” grew slower. This implied, from the pure reaction of joy and surprise, that more people were more pessimistic and started to think and reflect. One netizen directly stated in the comments: “Very sad. Who brought this virus?” However, people were still talking about hopes for the future; one audience member left a message: “Let us forget about politics for one moment, let's hope we make it through this.” Some audiences were even discussing the method to better prevent the virus with a comment: “If we were precautious this far spreading, we might have less in spreading [sic].” Some users discussed the lockdown in their own cities after watching the documentary and believed that it showed the determination the Chinese had to fight against the virus. Some even compared the procedure in their own countries, “Meanwhile in USA: come on, it's just a flu. If I get a flu, I will get over it.” “Trump: Nicely done people.” “When China imposes wearing of mask and lockdown, US said it was a violation of personal freedom and human rights.” This documentary not only allowed international audiences to have a more comprehensive understanding of the course of this health measure in Wuhan, China, but it also provided valuable experience as the epidemic spread throughout the world. Through this lockdown health measure, China proved a possible way of fighting the virus, and it surely increased the discussion of the lockdown policy globally.

In marketing, the use of big data can support the design of recommendation systems, to suggest, for instance, what a user might watch next on YouTube (Zhao et al., 2019). Among the studies on YouTube recommendation systems, there are studies on video content-based (Baluja et al., 2008), video main theme based (Bendersky et al., 2014), ItemCF-based (Davidson et al., 2010), view graph-based recommendation systems (Baluja et al., 2008), and deep learning solutions are also discussed (Covington et al., 2016). Our study suggested emotion analysis could enhance the recommendation system in the future.

## Conclusion

This article attempts to get the emotional responses and generate users' feedback from LDA topic modeling and emotion analysis on comments on the COVID-19 news documentary, which could support further user recommendations in video product marketing. This documentary on YouTube is called “The lockdown: One month in Wuhan”; we analyze global netizens' reactions to Wuhan's lockdown experience and the role of documentaries in health communication. According to the valid comments, we conclude that the major three themes, which account for 70% of all comments, are discussing stories in “fighting against the virus”, “medical workers are heroes”, and “showing respect.” In terms of emotion analysis, most of the netizens are quite positive. There was a peak in April 2020, and there was a growing trend of all emotions since July 2020, where “anger” and “disgust” grew faster, while “joy” and “surprise” grew slower. Most audiences are able to understand Wuhan's lockdown health measure and respect the selfless dedication of medical staff, but people are showing more pessimistic emotions and reflect on ways to cope with the virus, especially on the “lockdown” health measure. Generating users' feedback on Covid-19 news documentary comments enables us to understand more about people's responses to this video product; in addition, it could also support more effective user recommendations in the future for marketing purposes.

This paper has some limitations. We analyzed comments on the YouTube platform for a news documentary, but we only took into account text messages; more discussion in the form of images, snapshots, and short videos are not taken into consideration. This is because the YouTube platform provides limited information on users' feedback. Most YouTube videos have only implicit feedback (i.e., the user's comments or viewing behavior of the video) and a lack of explicit feedback (i.e., the user's rating of the video). This imposes limitations on our analysis, and we hope that future researchers can conduct experiments on this part of the data. Also, this research does not analyze the results of further marketing applications of sentiment data, but only discusses the application and future directions of it, such as user recommendation algorithms. Further discussion could be conducted with sentiment feedback analysis, applying user recommendation algorithms on larger datasets.

## Data Availability Statement

The original contributions presented in the study are included in the article/Supplementary Material, further inquiries can be directed to the corresponding author/s.

## Author Contributions

All authors listed have made a substantial, direct, and intellectual contribution to the work and approved it for publication.

## Funding

This research was supported in part by the Humanities and Social Sciences of the Ministry of Education Planning Fund (No. 21YJAZH073) and Jinan University, Guangzhou, China.

## Conflict of Interest

The authors declare that the research was conducted in the absence of any commercial or financial relationships that could be construed as a potential conflict of interest.

## Publisher's Note

All claims expressed in this article are solely those of the authors and do not necessarily represent those of their affiliated organizations, or those of the publisher, the editors and the reviewers. Any product that may be evaluated in this article, or claim that may be made by its manufacturer, is not guaranteed or endorsed by the publisher.
