# Supplementary material for: Users' Feedback on COVID-19 Lockdown Documentary: An Emotion Analysis and Topic Modeling Analysis
Source: Front Psychol. 2022 Jun 28;13:944049. doi: 10.3389/fpsyg.2022.944049 (PMC9275668; doi:10.3389/fpsyg.2022.944049)
Supplement: Supplementary file 1 [file Data_Sheet_1.docx]

Supplementary Material

# Supplementary Tables

TABLE I

Topic classification and keywords

| Theme Number | Theme Name | Theme Percentage | Topic number | Keywords | Topic Percentage |
| --- | --- | --- | --- | --- | --- |
| Theme 1 | Chinese fighting COVID-19 | 30.1 | Topic 4 | virus china corona countries well over america spread human new documentary everything stop against know down need first today take | 13.2 |
|  |  |  | Topic 3 | china chinese government love proud countries never being back media good strong western know through well life think even soon | 6.7 |
|  |  |  | Topic 9 | whole fight sad know china made stop watching make proud bless want coronavirus same fighting documentary human never virus strong | 10.2 |
| Theme 2 | About medical workers | 22.8 | Topic 5 | doctors nurses lives heroes care best back around hard respect thank take heart salute real virus work way life save | 13 |
|  |  |  | Topic 10 | medical workers please save others watch health situation salute doctors home pray love good lives being care need spread thanks | 9.8 |
| Theme 3 | Respect lockdown video | 19.8 | Topic 8 | lockdown bless respect india pandemic out 2020 good better real think best soon america today heart guys day through make | 12.7 |
|  |  |  | Topic 7 | video up life need even keep everything out way through make being watch love good sad better respect each disease | 7.1 |
| Theme 4 | Pray for safty | 14.2 | Topic 2 | safe virus pray strong together time fighting home work day come thank today please out fight each keep wuhan first | 14.2 |
| Theme 5 | Wuhan story | 6.6 | Topic 6 | wuhan great thanks city love china down thank guys time media western news want human documentary doctors watching here first | 6.6 |
| Theme 6 | About COVID-19 | 6.4 | Topic 1 | covid 19 always coronavirus chinese day against times italy disease here 2020 save health new doctors pandemic news lives made | 6.4 |

Remarks: The total percentage is 99.9% due to automatic rounding while exporting the results.

# Supplementary Figures

#
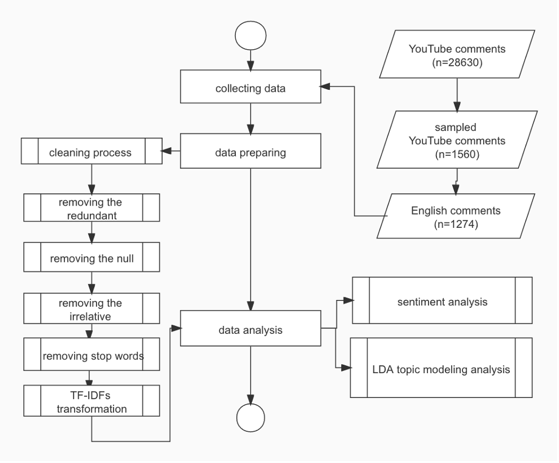


**Fig. 1.** Data processing flow chart


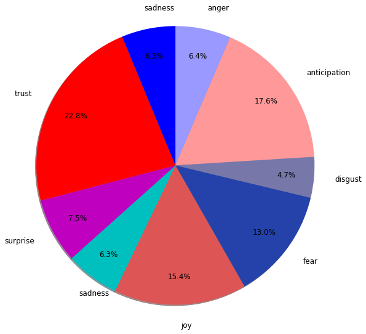


**Fig. 2.** Frequency ratio of emotional words


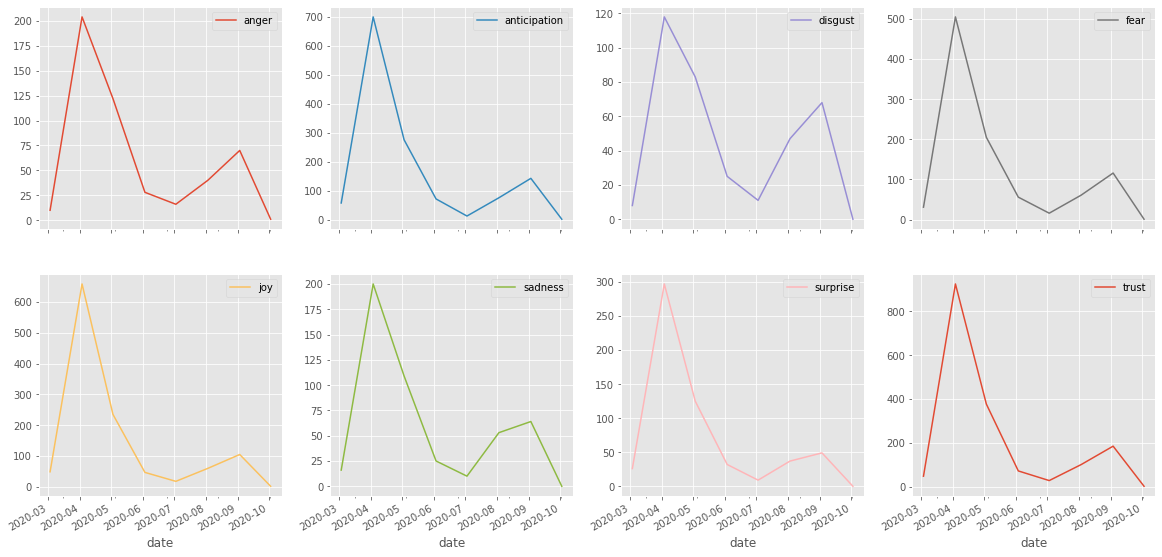


**Fig. 3.** Frequency ratio of emotional words


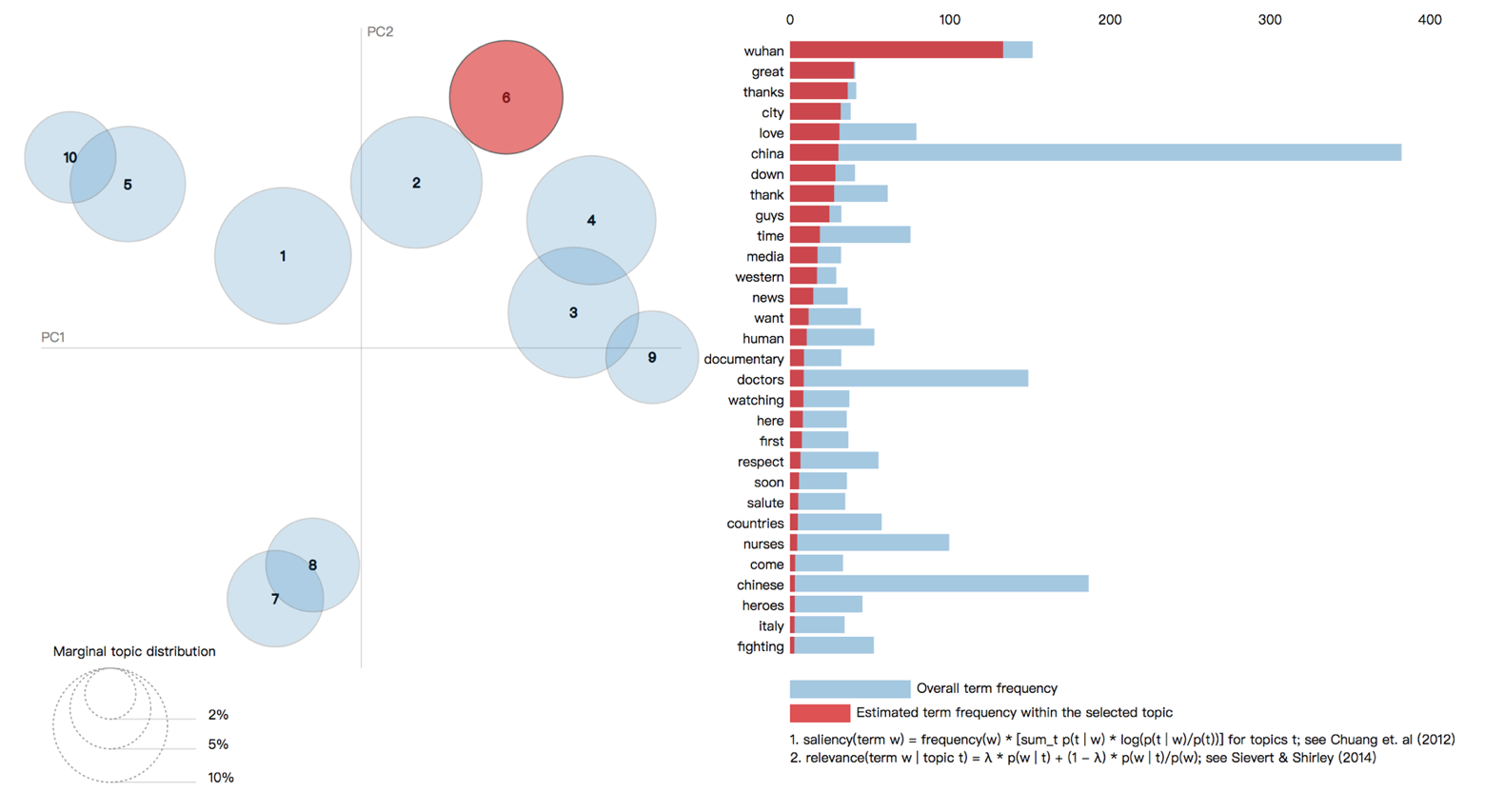


**Fig. 4.** Inter-topic distance map for Topic 6


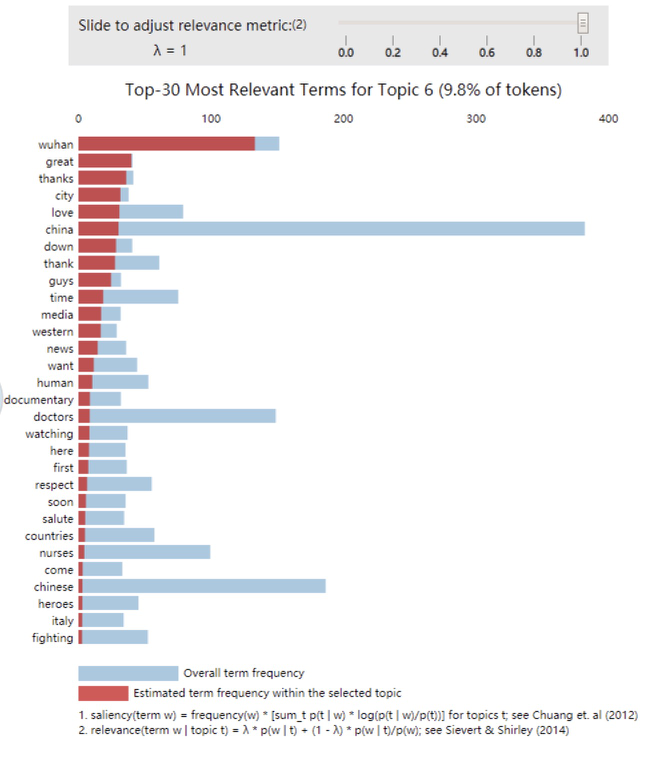


**Fig. 5.** Top-30 Most Relevant Terms for Topic 6 (9.8% of tokens)

**
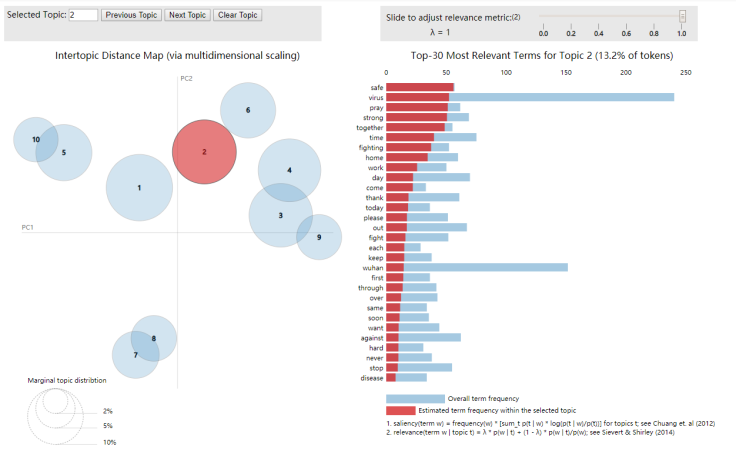
**

**Fig. 6.** Inter-topic distance map for Topic 2 &. Top-30 Most Relevant Terms for Topic 2 (13.2% of tokens
